# Discovery of Pancreatic Adenocarcinoma Biomarkers by Untargeted Metabolomics

**DOI:** 10.3390/cancers12041002

**Published:** 2020-04-18

**Authors:** Ariadna Martín-Blázquez, Cristina Jiménez-Luna, Caridad Díaz, Joaquina Martínez-Galán, Jose Prados, Francisca Vicente, Consolación Melguizo, Olga Genilloud, José Pérez del Palacio, Octavio Caba

**Affiliations:** 1 Fundación MEDINA, Centro de Excelencia en Investigación de Medicamentos Innovadores en Andalucía, 18016 Granada, Spain; ariadna.martin@medinaandalucia.es (A.M.-B.); caridad.diaz@medinaandalucia.es (C.D.); francisca.vicente@medinaandalucia.es (F.V.); olga.genilloud@medinaandalucia.es (O.G.); jose.perezdelpalacio@medinaandalucia.es (J.P.d.P.); 2 Department of Oncology, Ludwig Institute for Cancer Research, University of Lausanne, 1066 Epalinges, Switzerland; crisjilu@ugr.es; 3 Institute of Biopathology and Regenerative Medicine (IBIMER), Center of Biomedical Research (CIBM), University of Granada, 18016 Granada, Spain; melguizo@ugr.es (C.M.); ocaba@ugr.es (O.C.); 4 Service of Medical Oncology, Hospital Universitario Virgen de las Nieves, 18014 Granada, Spain; jmgalan22@hotmail.com; 5 Instituto Biosanitario de Granada (ibs. GRANADA), 18016 Granada, Spain

**Keywords:** metabolomics, pancreatic ductal adenocarcinoma, reverse-phase liquid chromatography, biomarker, diagnosis

## Abstract

Pancreatic ductal adenocarcinoma (PDAC) is one of the most aggressive and lethal cancers, with a 5-year survival rate of less than 5%. In fact, complete surgical resection remains the only curative treatment. However, fewer than 20% of patients are candidates for surgery at the time of presentation. Hence, there is a critical need to identify diagnostic biomarkers with potential clinical utility in this pathology. In this context, metabolomics could be a powerful tool to search for new robust biomarkers. Comparative metabolomic profiling was performed in serum samples from 59 unresectable PDAC patients and 60 healthy controls. Samples were analyzed by using an untargeted metabolomics workflow based on liquid chromatography, coupled to high-resolution mass spectrometry in positive and negative electrospray ionization modes. Univariate and multivariate analysis allowed the identification of potential candidates that were significantly altered in PDAC patients. A panel of nine candidates yielded excellent diagnostic capacities. Pathway analysis revealed four altered pathways in our patients. This study shows the potential of liquid chromatography coupled to high-resolution mass spectrometry as a diagnostic tool for PDAC. Furthermore, it identified novel robust biomarkers with excellent diagnostic capacities.

## 1. Introduction

Pancreatic ductal adenocarcinoma (PDAC) represents one of the deadliest neoplasms, with disturbingly close incidence and mortality rates. In 2017, there were 448,000 incident cases of pancreatic cancer (PC) globally, and the number of deaths had increased 2- to 3-fold, from 196,000 in 1990 to 441,000 [1]. Despite improvements in diagnosis and treatment and our growing understanding of the complex molecular events underlying PDAC, the overall 5-year survival rate remains worryingly low, at around 5% [2]. Currently, surgical resection is the only curative option for these patients, improving the 5-year survival rate from 5% to 25% [3]. However, given the lack of specific symptoms at early stages of this disease and its aggressive nature, a vast majority of patients are diagnosed with advanced cancer, when the tumor is unresectable [4]. This situation exposes the inadequacy of clinically available screening strategies to detect PDAC at an earlier and potentially curable stage. To date, the carbohydrate antigen 19-9 is the most widely used biomarker to monitor the recurrence and response to treatment in patients with elevated pretreatment levels; however, it is not indicated as a diagnostic tool for PDAC due to its low sensitivity and specificity [5]. Hence, the identification of more accurate, reliable, and cost-effective biomarkers for early PDAC diagnosis remains a challenge.

Metabolomics is the comprehensive study of metabolites, low molecular weight molecules that result from metabolic reactions and genetic/protein activity in a biological system [6]. Because the metabolome is closely related to the phenotype, metabolomics is increasingly proposed as a promising technology for biomarker discovery in cancer [7,8]. There is also growing research interest in mass spectrometry for metabolite analysis due to its high sensitivity, resolution, and reproducibility [9].

PDAC develops in a nutrition-deficient and hypoxic microenvironment characterized by poor angiogenesis and a large amount of dense connective tissue [10]. PC cells reprogram their metabolism to survive in these hostile conditions and maintain their elevated proliferation rate, which is not exhibited by normal cells [11]. Due to these changes, specific metabolites may appear in the patient’s serum that can be used as biomarkers. Previous studies have shown the value of metabolomics analyses to detect PDAC [12,13]. In 2012, Zhang et al. demonstrated the usefulness of the GlcA metabolite in blood for early diagnosis of PC [14]. Recently, 3-hydroxybutyrate, lactate, and dysregulated lipids in serum exosomes have also been proposed as early PDAC cancer biomarkers [15,16].

The present study adopted an untargeted metabolomics approach to identify potential biomarkers of PDAC. Serum samples were gathered from unresectable PDAC patients and healthy controls (HC) and analyzed by reverse-phase liquid chromatography (RPLC) coupled to high-resolution mass spectrometry (HRMS) in positive and negative electrospray ionization modes (ESI+, ESI-). After data treatment and statistical analysis, a clear distinction was observed between HCs and PDAC patients. Among the most discriminant metabolites, we tentatively identified 86 compounds that displayed significantly altered levels in PDAC serum samples.

## 2. Results

### 2.1. LC-HRMS Analysis

**Figure 1:**
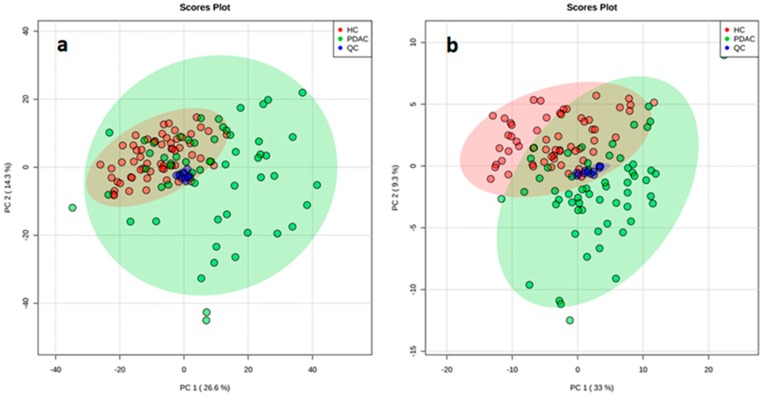
Principal component analysis score plots reveals a close clustering of the quality control (QC) samples by ESI+ (**a**) and ESI- (**b**) modes.

**Figure 2:**
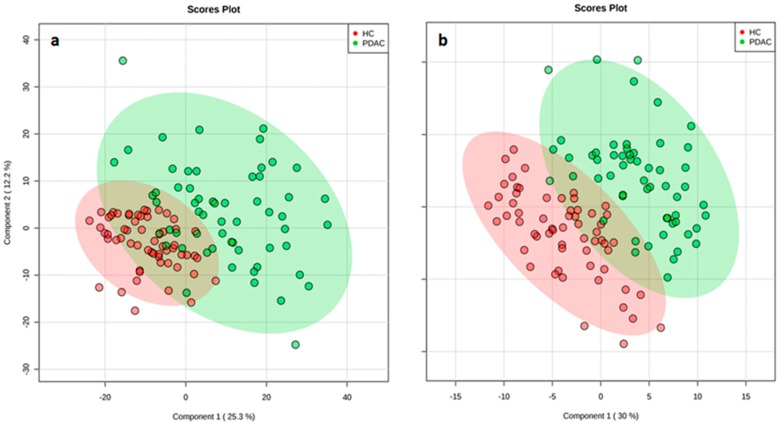
Partial least squares-discriminant analysis score plot shows a clear separation between the study groups (pancreatic ductal adenocarcinoma (PDAC) in green and healthy controls (HC) in red) in ESI+ (**a**) and ESI- (**b**) modes, suggesting that it is possible to discriminate between PDAC patients and HC.

RPLC, coupled to HRMS, was used in an untargeted metabolomics approach to detect candidate diagnostic markers for PDAC. Peaks obtained from RPLC-ESI+ and RPLC-ESI- experiments are summarized in Table 1. After alignment and filtering procedures, a data matrix of 1150 and 996 metabolite features was obtained for positive and negative ion modes, respectively. Only monoisotopic peaks (365 and 345 for positive and negative ions, respectively) were selected for further analysis. As a result of the Student’s *t*-test comparison between OS and study case samples, 220 and 113 features were removed as potential background and contaminants. At this point, the remaining features (145 and 152 for positive and negative ions, respectively) were assessed for reproducibility in QC samples. As a result, 61 and 18 features (in positive and negative ion mode, respectively) displayed coefficients of variation higher than 30% and were excluded. The remaining variables (84 and 134 for positive and negative ions, respectively) were evaluated in multivariate analysis. Instrumental repeatability was assessed using principal component analysis (PCA) score plots (Figure 1). The close clustering of QC samples indicated that the differences observed between HC and PDAC patients were only attributable to biological factors. In parallel, partial least squares-discriminant analysis score plots (Figure 2) illustrated a clear discrimination between the groups, with acceptable values of R^2^ and Q^2^ (Table 1). According to the established criteria, metabolomic data should display R^2^ ≥ 0.7 and Q^2^ ≥ 0.4 and should not vary by more than 0.2–0.3. No over-fitting was observed, indicating that these models successfully discriminated between HC and PDAC patients. The results of univariate statistical analysis (Student´s *t*-test or Wilcoxon rank-sum test) were applied to the global set of filtered variables (a total of 218 features, 84 from positive- and 134 from negative-ion analyses) yielding 154 variables with *p* < 0.05. Accordingly, 86 of these selected variables were tentatively identified by applying the criteria described in the following sections.

### 2.2. Pathway Analysis

Pathway analysis of the 86 tentatively identified variables revealed 17 deregulated metabolic pathways, and four of these pathways were statistically significant (*p* < 0.05) and might be potentially associated with unresectable PDAC (Table 2).

### 2.3. Biomarker Evaluation

**Figure 3:**
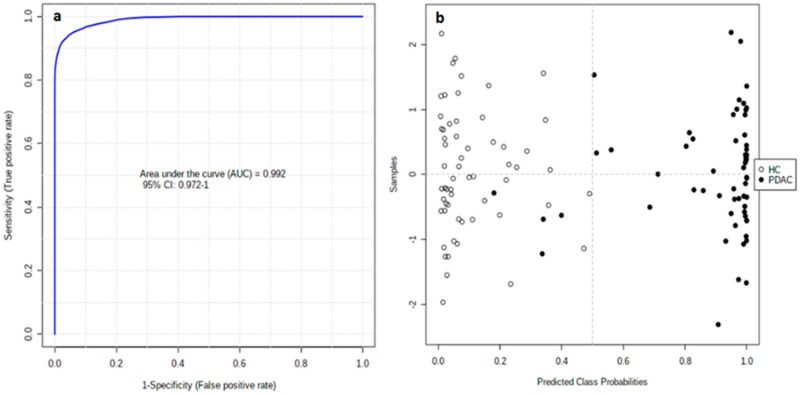
ROC curve for the nine-biomarker panel; 100 cross-validations were performed, and the results were averaged to generate the plot (**a**). Average of predicted class probabilities for each sample in 100 cross-validations. Given that the algorithm uses a balanced subsampling approach, the classification boundary is located at the center (x = 0.5, dotted line) (**b**).

The clinical value of all tentatively identified candidates (86 features showing *p* < 0.05 in univariate analysis) was evaluated by univariate receiver–operating characteristic curves (ROC) analysis (Table S1). As a result, 19 features displayed an area under the ROC curve (AUC) > 0.8. The individual markers were combined in a multivariate model to develop a more reliable algorithm (Figure S1). In order to avoid overfitting, the 19 features were cumulatively combined, starting with those with the highest individual AUC values, until a steady AUC value was achieved in the multivariate model. Accordingly, we propose a multivariate model based on nine candidate markers (Table 3) that discriminates unresectable PDAC patients from HC with an AUC value of 0.992 (95% CI of 0.972–1.000). The corresponding confusion matrix shows that all HC samples and 55 PDAC samples were correctly classified, while four PDAC samples were misclassified (Figure 3).

## 3. Discussion

There is an urgent need to improve diagnostic tools for PDAC, whose incidence is expected to increase worldwide over the next few years [17]. Using an untargeted metabolomics approach based on LC-HRMS and multivariate analysis, we identified a specific molecular signature for PDAC patients. Different omics technologies have been used to find robust and reliable biomarkers for different aspects of PDAC [18,19]. In the present study, we established a nine-biomarker panel with excellent diagnostic capacities for PDAC.

The conversion of cells from a normal to a cancerous state is accompanied by the reprogramming of metabolic pathways, an undisputed hallmark of cancer [20]. We detected four altered biological pathways in these patients: linoleic acid metabolism, glycerolipid metabolism, glycerophospholipid metabolism, and primary bile acid biosynthesis.

The symptoms of PDAC are highly varied and non-specific, usually appearing at more advanced stages of the disease [21], and there has been little metabolomic research on biomarkers for early PDAC. In one of the largest investigations to date, Fest et al. (2019), conducted a prospective study of pre-diagnostic samples from five biobanks; however, they concluded that analyses on a much larger scale were needed to obtain metabolomic biomarkers with an adequate predictive value to be clinically useful. Nevertheless, they proposed a panel of the metabolites showing the highest capacity to diagnose early PC, including docosahexaenoic acid. Interestingly, docosapentaenoic acid was altered in our cohort of patients with PDAC [22].

We highlight our finding that the levels of several phospholipids were altered in PDAC versus HC samples, most of which were downregulated in PDAC patients. A possible explanation for these results is the uncontrolled cell proliferation, which leads to phospholipid degradation in order to generate energy for expansion and dissemination [23]. This mechanism was previously proposed by Kühn et al. (2016) in a metabolomic study that demonstrated a correlation between elevated lysoPC (18:0) values and a lesser risk of breast, prostate, and colorectal cancer [24]. Concentrations of this metabolite were also higher in healthy controls than in our patients with PDAC. Similar results were observed in two previous studies of patients with PC. Thus, Fahrmann et al. used one patient cohort to search for biomarkers and another to construct the model, which was then validated in patients with early-stage PDAC. They proposed a panel of five metabolites to detect early-stage PDAC, including lysoPC (18:0) alongsideCA19.9, TIMP1, and LRG1, achieving an AUC of 0.924 [25]. Likewise, in the metabolomic study by Ritchie et al. of two ethnically and geographically diverse populations (USA Caucasian and Japanese), three of the metabolites found to be altered in patients with PC also differed between the patients with PDAC and HC in our study: lysoPC (18:0), lysoPC (18:2), and lysoPC (20:5) [26]. Another recent study of patients with PC also observed significantly lower values of lysoPC (18:2), alongsidePC-594 and two other serum-based choline metabolites, and this metabolite demonstrated 76.8% accuracy, 58.6% sensitivity, and 92.0% specificity for the diagnosis of PC [27]. Our results are also consistent with data recently published by Tao et al., who used untargeted lipidomic analysis in serum and exosomes and proposed a panel of 37 lipids differentially expressed by patients with PC versus HC [6]. The panel included three metabolites also proposed in our study as PDAC biomarkers: lysoPC (14:0), lysoPC (18:3), and lysoPE (20:5). In a previous study of populations in the USA and China, lysoPC (14:0) had also been included as a diagnostic biomarker in a panel of 30 metabolites that differed between patients with PC and HC [28]. Finally, we also observed decreased levels of the diacylglycerol CDP-DG (i-24:0/i-17:0) in PDAC patients. CDP-diacylglycerol is an important branchpoint intermediate in eukaryotic phospholipid biosynthesis and may be a key regulatory molecule in phospholipid metabolism; therefore, decreased levels of this molecule may explain the aforementioned reduction in glycerophospholipid levels [29].

Tumor cells upregulate the production of biosynthetic intermediates to build new cells, while increasing ATP levels and energy production. They use glucose, amino acids, and fatty acids as fuel sources, increasing the uptake of these metabolic substrates to meet increased demands for biosynthesis and energy production [10,30]. This may explain the decreased levels of phenylalanine in the serum of PDAC patients, as reported in various studies [31,32].

Highly proliferative cancer cells demonstrate a strong lipid avidity that is satisfied by increasing the uptake of exogenous lipids and lipoproteins [33], and we also detected alterations in the biosynthesis of fatty acids. Specifically, we detected reduced levels of TG (22:2), 3-oxooctadecanoic acid, and Oleoyl-L-carnitine, and increased levels of adrenic acid. Long-chain acyl-CoA synthetases are responsible for the activation of fatty acids and are commonly deregulated in cancer, which may explain the altered levels observed in these metabolites. We highlight that decreased Oleoyl-L-carnitine levels can cause cancer cachexia, a poor prognostic factor for PDAC [34,35].

Furthermore, significantly higher levels of the two bile acids, glycocholic acid and glycochenodeoxycholic acid 7-sulfate, were observed in PDAC patients, which may be attributable to tumor expansion into the bile duct. It has also been proposed that bile acid reflux into the pancreas can lead to malignant cell transformation by directly activating cancer signaling pathways [36,37]. Notably, the aforementioned study by Xie et al. (2015) in the USA and China identified glycocholic acid as the metabolite with the highest fold change value (3.65) in comparisons between 100 patients with PDAC and 100 healthy controlsin each population [28].

Two of the tentatively identified candidates were dehydroepiandrosterone sulfate (DHAS) and androsterone sulfate, which were decreased in PDAC patients. It has been shown that DHAS inhibits the growth of PC cells because it is a potent inhibitor of glucose-6 phosphate dehydrogenase, the rate-limiting enzyme of the hexose monophosphate shunt, a biochemical pathway that provides a substrate for DNA synthesis in neoplastic tissue [38,39].

One of the most discriminating markers was 4-oxo retinoic acid, which plays important roles in cell development and differentiation and in cancer treatment. Specifically, low and high doses of retinoic acid may cause cell cycle arrest and cancer cell apoptosis, respectively [40]. The increased levels detected in PDAC patients may therefore be a defense mechanism against tumor progression.

Besides their application for cancer diagnosis, metabolomic techniques have also proven useful to predict the response of patients to chemoradiation therapy. In one study in advanced rectal cancer patients, comparison of the lipodomic profile between responders and non-responders yielded a panel of five metabolites that predicted the response with an AUC of 0.95 and included lysoPC (16:0) [41]. Besides being identified in our study, lysoPC (16:0) was also proposed in a panel alongside lysoPC (20:4) for the predictive diagnosis, targeted prevention, and personalized treatment of pancreatic and biliary tract cancer, showing a sensitivity of 90.20% and specificity of 92.31% to discriminate between the two diseases [42].

Finally, 13-HODE levels were lower in PDAC patients than in HCs. This metabolite is regulated by the 15-Lipoxygenase-1 enzyme, which metabolizes linoleic acid to 13-HODE and loses its expression during tumor development in the human pancreas. According to these findings, loss of this enzyme may play an important role in pancreatic carcinogenesis, possibly as a tumor suppressor gene, consequently reducing the production of 13-HODE [43].

## 4. Materials and Methods

### 4.1. Sample Collection

The study included 119 samples: 60 from HC and 59 from unresectable PDAC patients. The baseline characteristics of all participants are detailed in Table 4. PDAC diagnosis was based on clinical evaluation and imaging studies and was histologically confirmed by surgery or imaging-guided biopsy. Blood samples from patients were obtained at Virgen de las Nieves Hospital in Granada (Spain) and the variation in different parameters regarding patients and sampling (fasting state, time of day of sampling, etc.) was minimized to obtain the maximum reproducibility in our study. In this way, fasting blood samples were drawn from the patients in hospital between 8 am and 9 am, before the initiation of any treatment. Written informed consent was obtained from all PDAC patients and HCs before their enrollment in the study, which was approved by the ethics committee of the hospital (Comité Ético de Investigación Provincial de Jaen; code: 1269-M1-19) and followed the principles of the Declaration of Helsinki.

Samples were collected in BD vacutainer SSTII advance tubes (Becton Dickinson, Franklin Lakes, NJ) with silica to activate clotting of the specimen. They were centrifuged for 10 min at 2450 rpm, and the supernatant was then aspirated and stored at −80 °C until analysis. Serum samples from HC were supplied by the Biobank of the Andalusian Public Health System and were obtained and treated in the same manner as the samples from patients.

### 4.2. Metabolite Extraction

All serum samples (75 µL) were kept at 4 °C throughout the analytical process. Proteins were removed using acetonitrile (AcN) (1:8 sample/AcN) and shaken for 2 min. Samples were then centrifuged at 15,200 rpm for 10 min at 4 °C. Supernatants were collected and transferred to HPLC analytical vials and evaporated in a GeneVac HT-8 evaporator (Savant, Holbrook, NY). Dry residues were reconstituted in 210 µL of AcN/water (50:50) with 0.1% formic acid and 250 ppb of internal standards. Finally, samples were shaken for 1 min and stored at −80 °C until their analysis.

### 4.3. LC-HRMS Analysis

Samples were analyzed using an Agilent 1290 LC system (Agilent Technologies, Santa Clara, CA, USA) coupled to a quadrupole-time-of-flight 5600 mass spectrometer (AB SCIEX Q-TOF 5600, Concord, ON, Canada) in ESI+ and ESI- modes.

In the ESI+ experiment, an Atlantis T3 HPLC C18 column (2.1 × 150 mm, 3 µm; Waters Corporation, Milford, MA, USA) kept at 25 °C was used for chromatographic separation. Mobile phases consisted of water:AcN (90:10) with 0.1% formic acid (eluent A) and AcN:water (90:10) with 0.1% formic acid (eluent B). The column was eluted with the following gradient: 0–0.5 min 1% eluent B; 0.5–11 min 99% eluent B; 11–15.50 min 99% eluent B; 15.50–15.60 min 1% eluent B and 15.60–20.00 min 1% eluent B. In the ESI- experiment, chromatographic separation was performed in a Gemini HPLC C18 column (100 mm × 2 mm, 3 µm; Phenomenex, California, USA) kept at 25 °C with mobile phases A water:AcN (90:10) and B AcN:water (90:10), which both contained 0.1% ammonia at 20%. The gradient was 0–0.3 min 1% eluent B; 0.3–7.3 min 99% eluent B, 7.3–10.3 min 99% eluent B, and 10.3–13.3 min 1% eluent B. In both experiments, the flow rate was 300 µL/min, and exact mass calibration was performed automatically every 10 injections. During the run sequence, 5 μL of serum samples were randomly injected, and quality control (QC) samples and organic solvent (OS) samples were analyzed every 10 and 30 injections, respectively.

The Q-TOF 5600 was operated using a TOF method, providing mass selection (80–1600 Da) with a high resolution in combination with information-dependent acquisition (IDA) and allowing fragmentation of the eight most intense ions to simultaneously collect full-scan HRMS and MS/MS information. Ion source parameters and IDA conditions were as follows: gas source 1: 50.00; gas source 2:50.00; curtain gas: 45.00; temperature: 500.00 °C; ionspray voltage floating: 4500.00; TOF masses: Min = 80.0000 Da Max = 1600.0000 Da; accumulation time: 0.2500 sec, IDA accumulation time: 0.1000 sec.

### 4.4. Data Set Creation

PeakView software (version 1.0 with Formula Finder plug-in version 1.0, AB SCIEX, Concord, ON) was used to evaluate the retention time (RT) and mass/charge (m/z) variability of the experiment. Then, MarkerView software (version 1.2.1, AB SCIEX, Concord, ON) was used to process raw LC-HRMS data, performing peak detection, alignment, and data filtering, and yielding a data matrix in which the measured m/z ratio, RT, and ion peak area were defined for each sample.

Data mining was performed by the program algorithm in the RT range of 1–17.20 min for the ESI+ experiment, and in the RT range of 1–13.00 min for the ESI- experiment. The peak intensity cutoff was set to 150 cps, and peak alignment was achieved using RT and m/z tolerances of 0.10 min and 5 ppm, respectively. A filter by “presence” was used to retain only masses that appeared in at least 12 samples in the study groups. Only monoisotopic peaks were considered in order to reduce mass redundancy and enhance selection of the true molecular feature. Next, we identified mass signals differentially expressed between the OS samples and case study samples (HC and PDAC), applying an additional filtering procedure with a *t*-test (*p* > 0.05) to remove background and contaminants, keeping the true biological mass signals from LC-HRMS data. Finally, we removed variables from the data matrix with unacceptable reproducibility (relative standard deviation > 30%) in QC samples. The following steps were carried out using Metaboanalyst 4.0 Web Server software.

### 4.5. Normalization and Analytical Validation

Probabilistic quotient normalization based on QC samples, log transformation, and Pareto scaling were used to convert the dataset to transform the data matrix into a more Gaussian-type distribution. Then, unsupervised PCA was carried out to evaluate the performance of the analytical system and detect possible outliers. In this regard, we evaluated the stability of the analytical system by plotting the QC samples on a PCA plot. The statistical quality of the model was assessed by goodness of fit (R^2^) and goodness of prediction (Q^2^). In parallel, the Hotelling T2 ellipses in a partial least squares-discriminant analysis score plot enabled the detection of outlier samples in both study groups. The elimination of outliers did not produce an increase in R^2^ or Q^2^ values.

### 4.6. Statistical Analysis

Filtered normalized variables in ion modes (ESI+ and ESI-) were combined in a single data matrix for global statistical analysis. The Shapiro–Wilk test was used to evaluate the normality of the data (*p* < 0.05). Next, the Student’s *t*-test or Wilcoxon-rank-sum test was applied to evaluate differences between patients with PDAC and HC. The Benjamin–Hochberg false discovery rate (FDR) correction for multiple comparisons was then performed to minimize the expected proportion of false positives (Type I errors). An FDR-corrected *p* value of 0.05 in the *t*-test is generally used in metabolomics as a cutoff threshold. Next, multivariate analysis was conducted to identify the variables responsible for the discrimination between groups.

### 4.7. Biomarker Evaluation

The clinical value of candidates was evaluated using their AUC values, performing univariate and multivariate ROC analyses to assess the clinical value of the candidates as biomarkers, either individually or in combination. The AUC values expressed in this study are flipped (1-AUC) and are, therefore, always presented as >0.5, independently of the case-control ratios.

### 4.8. Biomarker Identification

PeakView software (version 1.0 with Formula Finder plug-in version 1.0, AB SCIEX, Concord, ON) was used to estimate the elemental formula of selected marker compounds from accurate mass, isotopic clustering, and fragmentation patterns. Next, accurate mass queries were performed in compound databases (Metlin, Human Metabolome Database, Lipid Maps, PubChem, ChemSpider), and fragmentation patterns were searched in spectral databases (MassBank, NIST2014) for the tentative structural identification of the molecular formula. According to these criteria, all the metabolite identifications reported in our study should be considered tentative.

### 4.9. Pathway Analysis

Metaboanalyst 4.0 Web Server software was used to identify altered metabolic pathways. The metabolite ID matching was performed with the Human Metabolome Database. The analysis was adjusted by a hypergeometric test, and the impact on pathway topology was based on relative-betweenness centrality.

## 5. Conclusions

Our tentative identification of a molecular signature for PDAC patients highlights the potential of LC-HRMS untargeted metabolomics for the discovery of biomarkers. This study offers new insights into the molecular mechanism and signaling pathways of PDAC and proposes novel metabolic biomarkers that might be clinically relevant in the future. The main study limitation is that the patients were in advanced stages of PDAC at the time of their diagnosis; therefore, further research is needed to verify the usefulness of these markers for the diagnosis of patients with early-stage disease.

## Figures and Tables

**Table 1:** Extracted peaks from reverse-phase liquid chromatography (RPLC) positive and negative electrospray ionization modes (ESI+ and ESI-) high-resolution mass spectrometry (HRMS).

Ionization Mode	Total	Monoisotopics	After Organic Solvent (OS) Exclusion	RSD	Evaluated in PCA	R^2^	Q^2^
ESI+	1150	365	145	84	84	0.73	0.61
ESI-	966	345	152	134	134	0.73	0.66

Marker View software provided a data matrix with the extracted peaks. This software allowed the application of different filter steps to finally detect the features responsible for the discrimination between the groups. R^2^ and Q^2^ parameters from the partial least squares-discriminant analysis were used to assess model quality. RSD: relative standard deviation; PCA: principal component analysis.

**Table 2:** Altered pathways associated with PDAC.

Altered Pathways	*P ^1^*
Linoleicacidmetabolism	5.301 × 10^−3^
Glycerolipidmetabolism	5.664 × 10^−3^
Glycerophospholipidmetabolism	9.377 × 10^−3^
Primarybileacidbiosynthesis	2.190 × 10^−2^

Altered pathways obtained by Pathway Analysis using MetaboAnalyst 4.0. PDAC: pancreatic ductal adenocarcinoma. ^1^ Pathways with *p* < 0.05 were considered statistically significant.

**Table 3:** Selected candidate markers to create the proposed multivariate model.

ESImode	m/z	RT (min)	*p*(FDR)	PDAC/HC	AUC	Tentativeidentification
+	646.4145	7.65	1.65 × 10^−15^	↑	0.959	PS(12:0/15:1)
-	446.3760	9.44	3.75 × 10^−14^	↓	0.902	TG(22:2/15:0/18:3)
-	627.3741	3.53	7.46 × 10^−15^	↑	0.900	4-oxo-Retinoic acid
-	369.1747	3.29	2.71 × 10^−13^	↓	0.878	Androsterone sulfate
-	476.2792	5.08	4.65 × 10^−12^	↓	0.859	LysoPE(18:2)
-	311.1396	2.62	6.27 × 10^−17^	↓	0.858	Phenylalanylphenylalanine
+	430.2939	7.58	7.08 × 10^−10^	↑	0.850	all-trans-Decaprenyldiphosphate
+	1039.6721	10.79	1.73 × 10^−9^	↓	0.848	LysoPC(18:2)
-	367.1583	3.14	1.79 × 10^−9^	↓	0.847	Dehydroepiandrosterone sulfate

ESI: electrospray ionization; RT: retention time; FDR: false discovery rate; PDAC: pancreatic ductal adenocarcinoma patients; HC: healthy controls; AUC: area under the curve; PS: phosphatidylserine; TG: triglyceride; LysoPE: lysophosphatidylethanolamine; LysoPC: lysophosphatidylcholine. PDAC/HC ratio shows increased (↑) or decreased (↓) levels of each marker in PDAC group compared to HC and based in fold change ratio.

**Table 4:** Baseline characteristics of all study participants.

Characteristic	PDAC Patients	Healthy Controls
**Age (years ± SD)**	61.18 ± 12.17	56.16 ± 10.03
Sex		
Male	32	31
Female	27	29
**BMI (kg/m^2^ ± SD)**	25.40 ± 3.58	27.17 ± 3.76
**Pancreatitis**		
Yes	0	0
No	59	60
**Diabetes mellitus**		
No	45	60
Type I	0	0
Type II	14	0
Type IIIc	0	0
**Hypercholesterolemia**		
Yes	15	0
No	44	60
**Statin and/or fibrate intake**		
Yes	8	0
No	51	60
**Cachexia**		
Yes	0	0
No	59	60
**Previous surgeries**		
Yes	0	0
No	59	60
**Tumor stage**		
I	5	-
II	6	-
III	12	-
IV	36	-
**Site of primary tumor**		
Head	36	-
Body	15	-
Tail	8	-
**Icteric**		
Yes	18	-
No	41	-
**Metastatic**		
Yes	33	-
No	26	-
**Number of metastases**		
1	2	-
2	2	-
>2	29	-
**Metastatic site**		
Hepatic	26	-
Lymph node	11	-
Lung	7	-
Osseous	4	-
Brain	2	-
Other	15	-

## Supplementary Materials

The following are available online at https://www.mdpi.com/2072-6694/12/4/1002/s1, Table S1: Metabolites statistically significant with a tentative identification. Figure S1: ROC curve for the 19-biomarker panel; 100 cross-validations were performed, and the results were averaged to generate the plot (a). Average of predicted class probabilities for each sample in the 100 cross-validations. Given that the algorithm uses a balanced subsampling approach, the classification boundary is located at the center (x = 0.5, dotted line). The confusion matrix shows that all HC samples and 52 PDAC samples were correctly classified, but 4 PDAC samples were incorrectly classified (b).

## References

[1] Pourshams A., Sepanlou S.G., Ikuta K.S., Bisignano C., Safiri S., Roshandel G., Sharif M., Khatibian M., Fitzmaurice C., Nixon M.R. (2019). The global, regional, and national burden of pancreatic cancer and its attributable risk factors in 195 countries and territories, 1990–2017: A systematic analysis for the Global Burden of Disease Study 2017. Lancet Gastroenterol. Hepatol..

[2] McGuigan A., Kelly P., Turkington R.C., Jones C., Coleman H.G., McCain R.S. (2018). Pancreatic cancer: A review of clinical diagnosis, epidemiology, treatment and outcomes. World J. Gastroenterol..

[3] Satyananda V., Gupta R., Hari D.M., Yeh J., Chen K.T. (2019). Advances in Translational Research and Clinical Care in Pancreatic Cancer: Where Are We Headed?. Gastroenterol. Res. Pract..

[4] Brunner M., Wu Z., Krautz C., Pilarsky C., Grützmann R., Weber G.F. (2019). Current Clinical Strategies of Pancreatic Cancer Treatment and Open Molecular Questions. Int. J. Mol. Sci..

[5] Kleeff J., Korc M., Apte M., La Vecchia C., Johnson C.D., Biankin A.V., Neale R.E., Tempero M., Tuveson D.A., Hruban R.H. (2016). Pancreatic cancer. Nat. Rev. Dis. Primers.

[6] Schrimpe-Rutledge A.C., Codreanu S.G., Sherrod S.D., McLean J.A. (2016). Untargeted Metabolomics Strategies-Challenges and Emerging Directions. J. Am. Soc. Mass Spectrom..

[7] Roig B., Rodríguez-Balada M., Samino S., Lam E.W., Guaita-Esteruelas S., Gomes A.R., Correig X., Borràs J., Yanes O., Gumà J. (2017). Metabolomics reveals novel blood plasma biomarkers associated to the BRCA1-mutated phenotype of human breast cancer. Sci. Rep..

[8] Cheung P.K., Ma M.H., Tse H.F., Yeung K.F., Tsang H.F., Chu M.K.M., Kan C.M., Cho W.C.S., Ng L.B.W., Chan L.W.C. (2019). The applications of metabolomics in the molecular diagnostics of cancer. Expert Rev. Mol. Diagn..

[9] Miller R.A., Spellman D.S., Woods A.G., Darie C.C. (2014). Mass Spectrometry-Based Biomarkers in Drug Development. Advancements of Mass Spectrometry in Biomedical Research.

[10] Kamphorst J.J., Nofal M., Commisso C., Hackett S.R., Lu W., Grabocka E., Vander Heiden M.G., Miller G., Drebin J.A., Bar-Sagi D. (2015). Human pancreatic cancer tumors are nutrient poor and tumor cells actively scavenge extracellular protein. Cancer Res..

[11] Gu W., Tong Z. (2020). Clinical Application of Metabolomics in Pancreatic Diseases: A Mini-Review. Lab. Med..

[12] Morin A., Letouzé E., Gimenez-Roqueplo A.P., Favier J. (2014). Oncometabolites-driven tumorigenesis: From genetics to targeted therapy. Int. J. Cancer.

[13] Kobayashi T., Nishiumi S., Ikeda A., Yoshie T., Sakai A., Matsubara A., Izumi Y., Tsumura H., Tsuda M., Nishisaki H. (2013). A novel serum metabolomics-based diagnostic approach to pancreatic cancer. Cancer Epidemiol. Biomarkers Prev..

[14] Zhang L., Jin H., Guo X., Yang Z., Zhao L., Tang S., Mo P., Wu K., Nie Y., Pan Y. (2012). Distinguishing pancreatic cancer from chronic pancreatitis and healthy individuals by (1)H nuclear magnetic resonance-based metabonomic profiles. Clin. Biochem..

[15] Michálková L., Horník Š., Sýkora J., Habartová L., Setnička V. (2018). Diagnosis of pancreatic cancer via1H NMR metabolomics of human plasma. Analyst.

[16] Tao L., Zhou J., Yuan C., Zhang L., Li D., Si D., Xiu D., Zhong L. (2019). Metabolomics identifies serum and exosomes metabolite markers of pancreatic cancer. Metabolomics.

[17] Rawla P., Sunkara T., Gaduputi V. (2019). Epidemiology of Pancreatic Cancer: Global Trends, Etiology and Risk Factors. World J. Oncol..

[18] Rajamani D., Bhasin M.K. (2016). Identification of key regulators of pancreatic cancer progression through multidimensional systems-level analysis. Genome Med..

[19] Kwon M.S., Kim Y., Lee S., Namkung J., Yun T., Yi S.G., Han S., Kang M., Kim S.W., Jang J.Y. (2015). Integrative analysis of multi-omics data for identifying multi-markers for diagnosing pancreatic cancer. BMC Genom..

[20] Kato Y., Maeda T., Suzuki A., Baba Y. (2018). Cancer metabolism: New insights into classic characteristics. Jpn. Dent. Sci. Rev..

[21] Schmidt-Hansen M., Berendse S., Hamilton W. (2016). Symptoms of Pancreatic Cancer in Primary Care: A Systematic Review. Pancreas.

[22] Fest J., Vijfhuizen L.S., Goeman J.J., Veth O., Joensuu A., Perola M., Männistö S., Ness-Jensen E., Hveem K., Haller T. (2019). Search for Early Pancreatic Cancer Blood Biomarkers in Five European Prospective Population Biobanks Using Metabolomics. Endocrinology.

[23] Kamphorst J.J., Cross J.R., Fan J., de Stanchina E., Mathew R., White E.P., Thompson C.B., Rabinowitz J.D. (2013). Hypoxic and Ras-transformed cells support growth by scavenging unsaturated fatty acids from lysophospholipids. Proc. Natl. Acad. Sci. USA.

[24] Kühn T., Floegel A., Sookthai D., Johnson T., Rolle-Kampczyk U., Otto W., von Bergen M., Boeing H., Kaaks R. (2016). Higher plasma levels of lysophosphatidylcholine 18:0 are related to a lower risk of common cancers in a prospective metabolomics study. BMC Med..

[25] Fahrmann J.F., Bantis L.E., Capello M., Scelo G., Dennison J.B., Patel N., Murage E., Vykoukal J., Kundnani D.L., Foretova L. (2019). A Plasma-Derived Protein-Metabolite Multiplexed Panel for Early-Stage Pancreatic Cancer. J. Natl. Cancer Inst..

[26] Ritchie S.A., Akita H., Takemasa I., Eguchi H., Pastural E., Nagano H., Monden M., Doki Y., Mori M., Jin W. (2013). Metabolic system alterations in pancreatic cancer patient serum: Potential for early detection. BMC Cancer.

[27] Akita H., Ritchie S.A., Takemasa I., Eguchi H., Pastural E., Jin W., Yamazaki Y., Goodenowe D.B., Nagano H., Monden M. (2016). Serum Metabolite Profiling for the Detection of Pancreatic Cancer: Results of a Large Independent Validation Study. Pancreas.

[28] Xie G., Lu L., Qiu Y., Ni Q., Zhang W., Gao Y.T., Risch H.A., Yu H., Jia W. (2015). Plasma metabolite biomarkers for the detection of pancreatic cancer. J. Proteome Res..

[29] Frolkis A., Knox C., Lim E., Jewison T., Law V., Hau D.D., Liu P., Gautam B., Ly S., Guo A.C. (2010). SMPDB: The Small Molecule Pathway Database. Nucleic Acids Res..

[30] Hardie R.A., van Dam E., Cowley M., Han T.L., Balaban S., Pajic M., Pinese M., Iconomou M., Shearer R.F., McKenna J. (2017). Mitochondrial mutations and metabolic adaptation in pancreatic cancer. Cancer Metab..

[31] Urayama S., Zou W., Brooks K., Tolstikov V. (2010). Comprehensive mass spectrometry based metabolic profiling of blood plasma reveals potent discriminatory classifiers of pancreatic cancer. Rapid Commun. Mass Spectrom..

[32] Di Gangi I.M., Mazza T., Fontana A., Copetti M., Fusilli C., Ippolito A., Mattivi F., Latiano A., Andriulli A., Vrhovsek U. (2016). Metabolomic profile in pancreatic cancer patients: A consensus-based approach to identify highly discriminating metabolites. Oncotarget.

[33] Beloribi-Djefaflia S., Vasseur S., Guillaumond F. (2016). Lipid metabolic reprogramming in cancer cells. Oncogenesis.

[34] Kraft M., Kraft K., Gärtner S., Mayerle J., Simon P., Weber E., Schütte K., Stieler J., Koula-Jenik H., Holzhauer P. (2012). L-Carnitine-supplementation in advanced pancreatic cancer (CARPAN)—A randomized multicentre trial. Nutr. J..

[35] Tang Y., Zhou J., Hooi S.C., Jiang Y.M., Lu G.D. (2018). Fatty acid activation in carcinogenesis and cancer development: Essential roles of long-chain acyl-CoA synthetases. Oncol. Lett..

[36] Feng H.Y., Chen Y.C. (2016). Role of bile acids in carcinogenesis of pancreatic cancer: An old topic with new perspective. World J. Gastroenterol..

[37] Lindahl A., Heuchel R., Forshed J., Lehtiö J., Löhr M., Nordström A. (2017). Discrimination of pancreatic cancer and pancreatitis by LC-MS metabolomics. Metabolomics.

[38] Muscarella P., Boros L.G., Fisher W.E., Rink C., Melvin W.S. (1998). Oral Dehydroepiandrosterone Inhibits the Growth of Human Pancreatic Cancer in Nude Mice. J. Surg. Res..

[39] Melvin W.S., Boros L.G., Muscarella P., Brandes J.L., Johnson J.A., Fisher W.E., Schirmer W.J., Ellison E.C. (1997). Dehydroepiandrosterone-sulfate inhibits pancreatic carcinoma cell proliferation in vitro and in vivo. Surgery.

[40] Chen M.C., Hsu S.L., Lin H., Yang T.Y. (2014). Retinoic acid and cancer treatment. Biomedicine.

[41] Del Boccio P., Perrotti F., Rossi C., Cicalini I., Di Santo S., Zucchelli M., Sacchetta P., Genovesi D., Pieragostino D. (2017). Serum lipidomic study reveals potential early biomarkers for predicting response to chemoradiation therapy in advanced rectal cancer: A pilot study. Adv. Radiat. Oncol..

[42] Lee J.H., Yu S.E., Kim K.H., Yu M.H., Jeong I.H., Cho J.Y., Park S.J., Lee W.J., Han S.S., Kim T.H. (2018). Individualized metabolic profiling stratifies pancreatic and biliary tract cancer: A useful tool for innovative screening programs and predictive strategies in healthcare. EPMA J..

[43] Ding X.Z., Hennig R., Adrian T.E. (2003). Lipoxygenase and cyclooxygenase metabolism: New insights in treatment and chemoprevention of pancreatic cancer. Mol. Cancer.

