# Supplementary material for: Discovery of Pancreatic Adenocarcinoma Biomarkers by Untargeted Metabolomics
Source: Cancers (Basel). 2020 Apr 18;12(4):1002. doi: 10.3390/cancers12041002 (PMC7225994; doi:10.3390/cancers12041002)
Supplement: Supplementary file 1 [file cancers-12-01002-s001.zip › cancers-769330-supplementary/Supp.Material.docx]

**Supplementary Material**

|  | | Table 1. Statistically significant metabolites with tentative identification. | | | | | | | |
| --- | --- | --- | --- | --- | --- | --- | --- | --- | --- |
| **ESI** | **m/z** | | **RT (min)** | ***p*(FDR)** | **FC(PDAC/HC)** | **Cases/Controls** | **AUC** | **Molecular formula** | **ID** |
| - | 251.2021 | | 3.88 | 2.34E-06 | 2.36E+00 | ↑ | 0.764 | C16H28O2 | 7Z,10Z-Hexadecadienoic acid |
| - | 253.2196 | | 4.07 | 3.70E-04 | 1.77E+00 | ↑ | 0.700 | C16H30O2 | Palmitoleic acid |
| - | 255.2326 | | 7.94 | 2.56E-02 | 1.06E+00 | ↑ | 0.627 | C16H32O2 | Palmitic acid |
| - | 255.6305 | | 2.97 | 3.89E-02 | 2.47E+00 | ↑ | 0.602 | C26H43NO7S | Sulfolithocholylglycine |
| - | 263.6289 | | 2.79 | 5.81E-09 | 1.25E+01 | ↑ | 0.835 | C26H43NO8S | Glycochenodeoxycholic acid 7-sulfate |
| - | 279.2343 | | 4.26 | 8.13E-04 | 1.71E+00 | ↑ | 0.693 | C18H32O2 | Linoleic acid |
| - | 281.2484 | | 4.41 | 8.98E-04 | 1.58E+00 | ↑ | 0.691 | C18H34O2 | Vaccenic acid |
| - | 283.2645 | | 4.51 | 2.81E-02 | 1.52E+00 | ↑ | 0.632 | C18H36O2 | Stearic acid |
| - | 288.6192 | | 2.82 | 6.42E-07 | 5.07E+01 | ↑ | 0.786 | C26H45NO9S2 | Taurochenodeoxycholate-7-sulfate |
| - | 295.2289 | | 3.65 | 5.87E-04 | 1.01E+00 | ↑ | 0.724 | C18H32O3 | 12,13-EpOME |
| - | 297.2432 | | 4.36 | 1.95E-03 | 1.68E+00 | ↑ | 0.679 | C18H36O4 | MG(15:0) |
| - | 305.2515 | | 4.36 | 3.97E-03 | 1.79E+00 | ↑ | 0.662 | C20H34O2 | 5,8,11-Eicosatrienoic acid |
| - | 307.2641 | | 4.54 | 1.28E-07 | 2.12E+00 | ↑ | 0.798 | C20H36O2 | 8Z,11Z-eicosadienoic acid |
| - | 311.1396 | | 2.62 | 6.27E-17 | 3.88E-01 | ↓ | 0.858 | C18H20N2O3 | Phenylalanylphenylalanine |
| - | 311.2228 | | 4.21 | 3.91E-03 | 1.68E+00 | ↑ | 0.641 | C18H32O4 | 9(S)-HPODE |
| - | 313.2401 | | 4.21 | 2.74E-03 | 1.68E+00 | ↑ | 0.656 | C18H34O4 | 9,10-DiHOME |
| + | 316.2482 | | 7.16 | 1.57E-04 | 1.39E+00 | ↑ | 0.720 | C17H33NO4 | Decanoylcarnitine |
| - | 329.2501 | | 4.36 | 1.50E-06 | 1.95E+00 | ↑ | 0.760 | C22H34O2 | Docosapentaenoic acid |
| - | 331.2658 | | 4.52 | 6.31E-10 | 2.21E+00 | ↑ | 0.837 | C22H36O2 | Adrenic acid |
| - | 337.3096 | | 4.99 | 1.13E-02 | 1.98E+00 | ↑ | 0.627 | C22H42O2 | Erucic acid |
| - | 367.1583 | | 3.14 | 1.79E-09 | 4.10E-01 | ↓ | 0.847 | C19H28O5S | Dehydroepiandrosterone sulfate |
| - | 369.1761 | | 2.99 | 3.49E-08 | 4.57E-01 | ↓ | 0.790 | C19H30O5S | Epiandrosterone sulfate |
| - | 369.1747 | | 3.29 | 2.71E-13 | 3.10E-01 | ↓ | 0.878 | C19H30O5S | Androsterone sulfate |
| + | 370.2963 | | 8.55 | 1.86E-04 | 1.38E+00 | ↑ | 0.714 | C21H39NO4 | trans-2-Tetradecenoylcarnitine |
| - | 387.2602 | | 4.21 | 5.29E-04 | 1.78E+00 | ↑ | 0.692 | C45H77O8P | PA(22:2/20:4) |
| - | 389.2744 | | 4.45 | 5.49E-04 | 1.69E+00 | ↑ | 0.699 | C45H81O8P | PA(20:3/22:1) |
| - | 390.2981 | | 8.53 | 4.70E-02 | 1.02E+00 | ↑ | 0.610 | C22H43NO3 | N-stearoyl GABA |
| - | 391.2856 | | 3.17 | 1.57E-02 | 1.15E+00 | ↑ | 0.613 | C24H40O4 | Hyodeoxycholic acid |
| - | 391.2847 | | 3.49 | 4.68E-02 | 1.60E+00 | ↑ | 0.593 | C24H40O4 | Murocholic acid |
| - | 399.2175 | | 4.38 | 1.97E-02 | 1.58E+00 | ↑ | 0.635 | C21H32O3 | 17a-Hydroxypregnenolone |
| + | 400.3427 | | 9.93 | 8.09E-08 | 1.47E+00 | ↑ | 0.791 | C23H45NO4 | L-Palmitoylcarnitine |
| - | \| 413.1930 \| \| --- \| | | 5.93 | 3.53E-02 | 9.51E-01 | ↓ | 0.621 | C16H33O7P | LysoPA(a-13:0/0:0) |
| + | 424.3420 | | 9.59 | 1.57E-06 | 1.42E+00 | ↑ | 0.776 | C25H45NO4 | Linoleyl carnitine |
| + | 426.3587 | | 10.13 | 1.39E-09 | 1.71E+00 | ↑ | 0.822 | C25H47NO4 | Oleoyl L-carnitine |
| - | 429.3010 | | 3.63 | 6.48E-04 | 9.00E-01 | ↓ | 0.716 | C27H42O4 | 7alpha-Hydroxy-3-oxo-4-cholestenoate |
| + | 430.2939 | | 7.58 | 7.08E-10 | 8.48E+00 | ↑ | 0.850 | C50H84O7P2 | all-trans-Decaprenyl diphosphate |
| + | 437.1929 | | 9.63 | 4.41E-03 | 1.17E+00 | ↑ | 0.647 | C16H33O7P | LysoPA(a-13:0/0:0) |
| - | 446.3760 | | 9.44 | 3.75E-14 | 4.33E-01 | ↓ | 0.902 | C58H102O6 | TG(22:2/15:0/18:3) |
| - | 449.2528 | | 3.75 | 1.25E-03 | 6.70E-01 | ↓ | 0.686 | C25H40O8 | 17-hydroxyandrostane-3-glucuronide |
| + | 450.3229 | | 8.95 | 8.84E-03 | 1.87E+00 | ↑ | 0.632 | C26H43NO5 | Glycodeoxycholic acid |
| - | 452.2744 | | 5.16 | 2.49E-03 | 9.22E-01 | ↓ | 0.670 | C21H44NO7P | LysoPE(16:0) |
| - | 464.3020 | | 3.33 | 5.56E-05 | 1.61E+01 | ↑ | 0.732 | C26H43NO6 | Glycocholic acid |
| - | 465.3024 | | 5.14 | 2.68E-02 | 1.62E+00 | ↑ | 0.664 | C27H46O4S | Cholesterol sulfate |
| + | 466.3148 | | 7.54 | 2.01E-08 | 1.41E+01 | ↑ | 0.822 | C26H43NO7 | Glycocholic acid |
| + | 468.3056 | | 9.58 | 1.57E-06 | 6.58E-01 | ↓ | 0.777 | C22H46NO7P | LysoPC(14:0) |
| + | 468.3078 | | 9.84 | 3.32E-06 | 6.60E-01 | ↓ | 0.747 | C22H46NO7P | LysoPE(17:0) |
| - | 476.2792 | | 5.08 | 4.65E-12 | 5.95E-01 | ↓ | 0.859 | C23H44NO7P | LysoPE(18:2) |
| + | 478.2883 | | 10.45 | 6.17E-09 | 5.57E-01 | ↓ | 0.806 | C23H44NO7P | LysoPE(18:2) |
| + | 478.2934 | | 10.65 | 1.66E-06 | 5.73E-01 | ↓ | 0.775 | C23H41O7P | LysoPA(20:3) |
| - | 478.2946 | | 5.27 | 1.41E-09 | 6.28E-01 | ↓ | 0.814 | C23H46NO7P | LysoPE(18:1) |
| + | 496.3399 | | 11.05 | 5.03E-03 | 8.12E-01 | ↓ | 0.675 | C24H50NO7P | LysoPC(16:0) |
| - | 498.2885 | | 3.58 | 2.28E-02 | 4.79E+00 | ↑ | 0.617 | C26H45NO6S | Taurodeoxycholic acid |
| - | 498.2623 | | 4.78 | 1.53E-06 | 6.30E-01 | ↓ | 0.777 | C25H42NO7P | LysoPE(20:5) |
| - | 500.2798 | | 5.05 | 1.87E-06 | 9.91E-01 | ↓ | 0.752 | C25H44NO7P | LysoPE(20:4) |
| + | 508.3765 | | 12.28 | 1.94E-04 | 1.13E+00 | ↑ | 0.720 | C26H54NO6P | LysoPC(P-18:0) |
| + | 517.3308 | | 9.66 | 1.33E-06 | 4.13E+00 | ↑ | 0.748 | C26H45NO6S | Tauroursodeoxycholic acid |
| + | 518.3234 | | 10.14 | 1.11E-05 | 6.78E-01 | ↓ | 0.753 | C26H48NO7P | LysoPC(18:3) |
| + | 520.3427 | | 10.75 | 8.09E-08 | 6.30E-01 | ↓ | 0.795 | C27H54NO6P | PC(P-19:1) |
| - | 524.2771 | | 5.01 | 2.22E-02 | 1.27E+00 | ↑ | 0.639 | C27H44NO7P | LysoPE(22:6) |
| + | 524.3705 | | 13.27 | 1.99E-02 | 7.60E-01 | ↓ | 0.632 | C26H54NO7P | LysoPC(18:0) |
| + | 526.2932 | | 10.65 | 3.02E-02 | 1.12E+00 | ↑ | 0.585 | C27H44NO7P | LysoPE(22:6) |
| - | 537.4900 | | 4.36 | 7.38E-04 | 3.00E+00 | ↑ | 0.690 | C37H74NO8P | PE(16:0/16:0) |
| + | 542.3230 | | 10.75 | 2.30E-08 | 6.28E-01 | ↓ | 0.800 | C28H48NO7P | LysoPC(20:5) |
| - | 559.4757 | | 4.24 | 2.50E-03 | 5.06E+00 | ↑ | 0.670 | C36H66O5 | DG(18:2/15:0) |
| - | 563.5088 | | 4.45 | 2.42E-04 | 3.81E+00 | ↑ | 0.699 | C36H70O5 | DG(13:0/i-20:0) |
| + | 568.3385 | | 10.71 | 4.71E-02 | 1.08E+00 | ↑ | 0.597 | C30H50NO7P | LysoPC(22:6) |
| - | 575.4694 | | 3.98 | 2.73E-05 | 7.29E-01 | ↓ | 0.745 | C36H64O5 | DG(15:0/0:0/18:3) |
| - | 585.4878 | | 4.45 | 2.84E-04 | 2.72E+00 | ↑ | 0.700 | C38H68O5 | DG(20:3/15:0/0:0) |
| - | 591.4610 | | 3.7 | 3.21E-08 | 5.18E-01 | ↓ | 0.803 | C18H32O3 | 13-HODE |
| - | 595.4906 | | 3.97 | 4.92E-08 | 4.99E-01 | ↓ | 0.813 | C18H34O3 | 3-Oxooctadecanoic acid |
| - | 605.4060 | | 5.16 | 1.10E-04 | 8.67E-01 | ↓ | 0.723 | C15H29NO5 | 3-hydroxyoctanoyl carnitine |
| - | 607.4728 | | 4.26 | 1.57E-03 | 2.62E+00 | ↑ | 0.665 | C40H66O5 | DG(22:6/15:0/0:0) |
| - | 619.2933 | | 4.23 | 3.55E-04 | 1.76E+00 | ↑ | 0.706 | C29H49O12P | 1-Arachidonoyl-sn-glycero-3-phospho-(1'-myo-inositol) |
| - | 627.3741 | | 3.53 | 7.46E-15 | 1.04E+01 | ↑ | 0.900 | C20H26O3 | 4-oxo-Retinoic acid |
| - | 641.3060 | | 4.45 | 2.69E-05 | 1.80E+00 | ↑ | 0.727 | C33H54O7 | Cholesterol glucuronide |
| + | 646.4145 | | 7.65 | 1.65E-15 | 9.43E+00 | ↑ | 0.959 | C33H62NO10P | PS(12:0/15:1) |
| - | 698.4121 | | 7.81 | 7.84E-05 | 7.52E-01 | ↓ | 0.710 | C35H68NO8P | PE(14:1/16:0) |
| - | 698.4088 | | 8.00 | 3.21E-06 | 6.72E-01 | ↓ | 0.745 | C36H62NO10P | PS(18:4/12:0) |
| - | 698.4152 | | 8.01 | 2.34E-06 | 6.56E-01 | ↓ | 0.747 | C35H68NO8P | PE(16:0/14:1) |
| - | 736.3974 | | 7.98 | 2.54E-06 | 7.13E-01 | ↓ | 0.743 | C36H64NO10P | PS(12:0/18:3) |
| - | 745.4118 | | 7.99 | 1.19E-05 | 7.03E-01 | ↓ | 0.715 | C34H68O13P2 | PGP(i-13:0/a-15:0) |
| - | 759.4340 | | 7.97 | 1.69E-05 | 7.46E-01 | ↓ | 0.712 | C41H71O8P | PA(20:3/18:2) |
| + | 991.6758 | | 11.33 | 2.35E-02 | 8.92E-01 | ↓ | 0.631 | C21H19I4NO10 | Thyroxine glucuronide |
| + | 1002.6570 | | 11.33 | 1.03E-02 | 8.34E-01 | ↓ | 0.637 | C55H98NO10P | PE-NMe2(13D5/13D5) |
| + | 1013.6480 | | 11.32 | 8.77E-03 | 7.60E-01 | ↓ | 0.638 | C51H94N2O16 | NeuAcα2-3Galβ-Cer(d18:1/16:0) |
| + | 1039.6721 | | 10.79 | 1.73E-09 | 4.07E-01 | ↓ | 0.848 | C26H50NO7P | LysoPC(18:2) |
| + | 1041.6883 | | 11.33 | 2.76E-02 | 8.56E-01 | ↓ | 0.627 | C25H52NO7P | LysoPC(17:0) |
| + | 1043.6978 | | 11.77 | 2.68E-02 | 8.94E-01 | ↓ | 0.606 | C26H52NO7P | LysoPC(18:1) |
| + | 1061.6462 | | 10.79 | 9.42E-09 | 4.01E-01 | ↓ | 0.829 | C25H50NO9P | PS(19:0) |
| + | 1062.6503 | | 10.79 | 2.33E-09 | 3.79E-01 | ↓ | 0.844 | C53H99N3O15P2 | CDP-DG(i-24:0/i-17:0) |

Candidates were selected according to the corresponding test (FDR; *p*< 0.05). Their potential as clinical biomarkers was evaluated using ROC curves. Accurate mass and MS/MS patterns allowed tentative identification of the molecular formula. Fold change is expressed as the ratio between the two averages (PDAC/HC). LysoPA: lysophosphatidic acid; LysoPC: lysophosphatidylcholine; LysoPE: lysophosphatidylethanolamine; PC: phosphatidylcholine; PS: phosphatidylserine; PE-NMe2: dimethylphosphatidylethanolamine; CDP-DG: cytidine diphosphate diacylglycerol; MG: monoacylglycerol; PA: phosphatidic acid; DG: diacylglycerol; PE: phosphatidylethanolamine; PGP: phosphatidylcylerolphosphate; TG: triacylglycerol; ↓: metabolite decreased in PDAC patients; ↑: metabolite increased in PDAC patients.


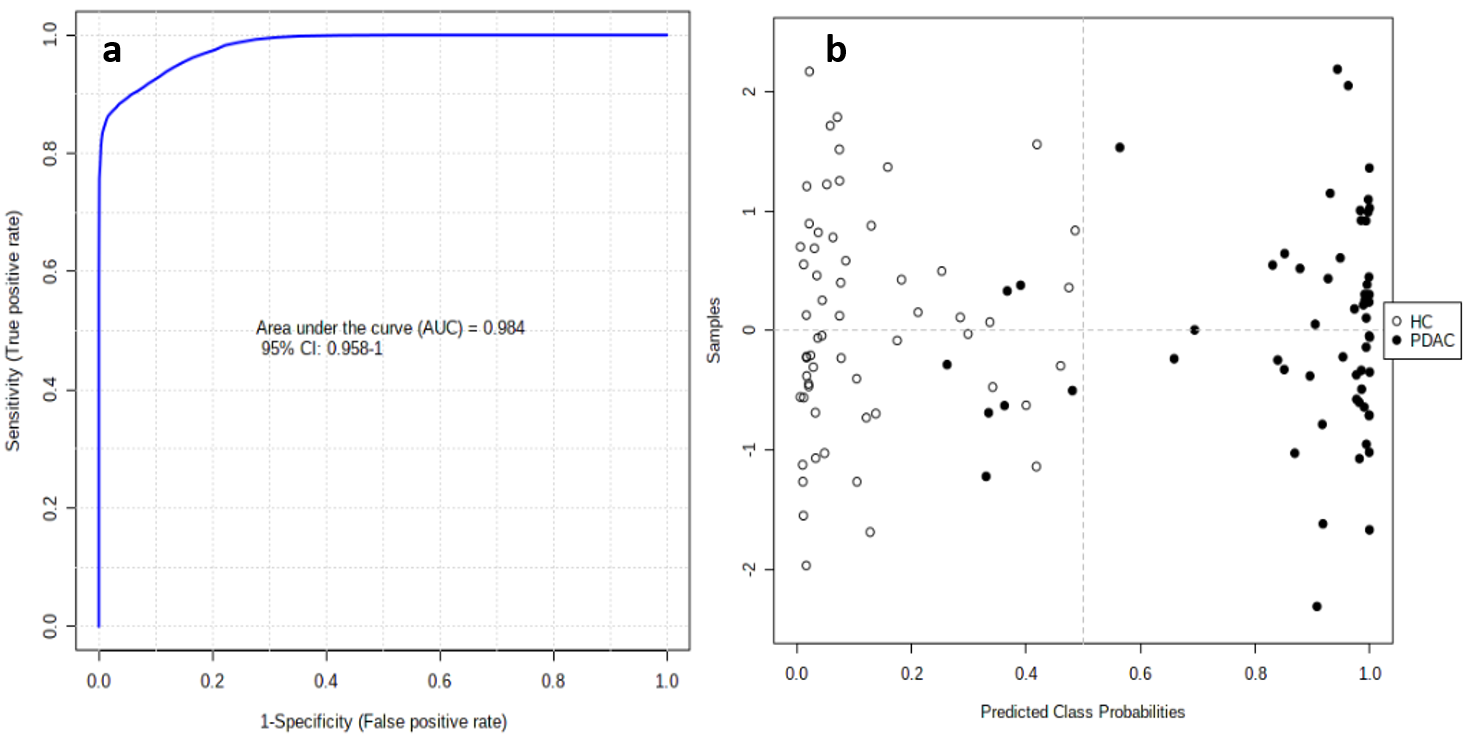


**Supplementary Figure 1.** ROC curve for the 19-biomarker panel; 100 cross-validations were performed, and the results were averaged to generate the plot (a).Average of predicted class probabilities for each sample in the 100 cross-validations. Given that the algorithm uses a balanced subsampling approach, the classification boundary is located at the center (x = 0.5, dotted line). The confusion matrix shows that all HC samples and 52 PDAC samples were correctly classified, but 4 PDAC samples were incorrectly classified(b).
